# Genomic Diversity and Extended‐Spectrum *β*‐Lactamase Gene Contexts of Community Resident–Carried *Escherichia coli* in Ecuador

**DOI:** 10.1155/ijm/1377880

**Published:** 2026-09-01

**Authors:** Judith Dzifa Azumah, Hoa Thi Thanh Hoang, Yen Hai Le, Manuel Calvopina, Carlos Bastidas-Caldes, Mayumi Yamamoto, Yoshimasa Yamamoto

**Affiliations:** ^1^ The United Graduate School of Drug Discovery and Medical Information Sciences, Gifu University, Gifu, Japan, gifu-u.ac.jp; ^2^ Faculty of Medical Technology, Hanoi Medical University, Hanoi, Vietnam, hmu.edu.vn; ^3^ One Health Research Group, Universidad de Las Américas, Quito, Ecuador, udla.edu.ec; ^4^ School of Medicine, Universidad Espíritu Santo, Samborondón, Ecuador, uees.me; ^5^ Health Administration Center, Gifu University, Gifu, Japan, gifu-u.ac.jp

**Keywords:** community carriage, CTX-M *β*-lactamases, Ecuador, ESBL-producing *Escherichia coli*, plasmid-mediated antimicrobial resistance, whole-genome sequencing

## Abstract

Community carriage of extended‐spectrum *β*‐lactamase (ESBL)‐producing *Escherichia coli* represents an important reservoir of antimicrobial resistance. However, the genomic diversity and population structure of ESBL‐producing *E. coli* circulating in community settings remain poorly characterized. This study aimed to characterize ESBL‐producing *E. coli* isolated from fecal samples of residents in Ecuador, with an emphasis on the diversity and genomic context of ESBL genes. ESBL‐producing *E. coli* was isolated from fecal samples obtained from 55 residents using MacConkey agar supplemented with cefotaxime. Whole‐genome sequencing of the isolates was performed using a hybrid approach combining long‐ and short‐read platforms. Plasmids and *β*‐lactamase genes were identified using DFAST and PlasmidFinder. Bacterial identification and antimicrobial susceptibility testing were conducted by MALDI‐TOF MS and the broth microdilution method, respectively. ESBL‐producing *E. coli* were isolated from 35 of 55 fecal samples (63.6%). Complete circular genomes were obtained from 31 isolates. All isolates harbored *bla*_CTX-M_ genes, predominantly belonging to the *bla*_CTX-M-1_ group, whereas 65.7% carried *bla*_TEM_, mainly *bla*_TEM-1_, and related variants. Although *β*‐lactamase genes were predominantly plasmid‐borne, chromosomal integration was detected in 40% of the isolates. Notably, 87.5% of the isolates harbored IncF plasmids with multiple replicons. Conserved IS*26*‐flanked transposons carrying *bla*_CTX-M_ and *bla*_TEM_ were frequently identified in the plasmids. Phylogenetic analysis revealed substantial genomic diversity across seven phylogroups, together with closely related isolates detected within and between households. These findings provide high‐resolution genomic insights into the ESBL determinants circulating in community residents and reveal region‐specific patterns of ESBL genomic diversity.

## 1. Introduction

Extended‐spectrum *β*‐lactamase (ESBL)‐producing *Escherichia coli* have emerged as a major global public health concern, contributing to the burden of antimicrobial resistance (AMR) [1, 2]. Over the past two decades, ESBL‐producing *E. coli* have transitioned from healthcare‐associated pathogens to widely disseminated organisms in the community, with increased detection among healthy individuals lacking recent healthcare exposure [3, 4]. Community carriage of ESBL‐producing *E. coli* is recognized as a critical precursor of infection and a key driver of sustained transmission at the population level [5].

Globally, nonclinical human studies have consistently shown that intestinal microbiomes with ESBL‐producing *E. coli* are common in low‐ and middle‐income countries, where the prevalence rates often exceed those reported in high‐income regions [6, 7]. In Latin America, fecal carriage studies among community residents, particularly children and household contacts, have documented high ESBL prevalence, highlighting the importance of the human gut as a reservoir for resistant *E. coli* outside of hospital environments [8–10]. However, many of these investigations have primarily emphasized prevalence, clonal diversity, or transmission dynamics, with less focus on the characterization of the underlying ESBL gene repertoire maintained within the intestinal microbiome.

From a genomic perspective, ESBL‐mediated resistance in *E. coli* is primarily driven by CTX‐M‐type enzymes, which have replaced TEM‐ and SHV‐derived ESBLs worldwide [11]. CTX‐M variants such as CTX‐M‐15, CTX‐M‐55, CTX‐M‐27, and CTX‐M‐2 have been repeatedly associated with multidrug‐resistant phenotypes because of their frequent colocalization with additional resistance determinants, including genes conferring resistance to fluoroquinolones, aminoglycosides, and sulfonamides [12–14]. Although the _TEM_ family alleles typically exhibit limited intrinsic extended‐spectrum activity, they are commonly detected alongside CTX‐M genes in mobile genetic elements, contributing to the persistence of resistance through coselection [15].

The rapid and sustained spread of ESBL genes is largely driven by horizontal gene transfer mediated by plasmids, particularly those belonging to the IncF incompatibility group, which are well adapted to *E. coli* and exhibit high stability in the intestinal environment [16, 17]. These plasmids facilitate the accumulation and maintenance of multiple resistance genes and are efficiently transmitted within dense microbial communities such as the human gut [18, 19]. Additional factors promoting dissemination include close human–animal–environment contact, inadequate sanitation, widespread antimicrobial use, and selective pressures exerted by commonly used antibiotics in both clinical and nonclinical settings [20].

In Ecuador, genomic studies of ESBL‐producing *E. coli* from resident fecal samples and household environments have reported the frequent detection of *bla*_CTX-M-15_ and *bla*_CTX-M-55_, together with extensive strain sharing between humans, domestic animals, and environmental sources [8]. Parallel investigations of food, livestock, and surface water have identified overlapping *bla*_CTX-M_ variants, including *bla*_CTX-M-15_, *bla*_CTX-M-55_, and *bla*_CTX-M-65_, supporting the One Health framework for ESBL dissemination [21]. Despite this growing body of evidence, few studies have systematically characterized the full diversity of ESBL genes present in fecal *E. coli* isolates from residents, limiting our understanding of how resistance is structured and maintained within the intestinal microbiome.

Addressing this gap is critical, as the resident intestinal microbiome represents a stable ecological niche where ESBL genes can persist, diversify, and serve as a source for onward transmission or infection. Therefore, this study aimed to characterize ESBL‐producing *E. coli* isolated from fecal samples of healthy residents in Ecuador, focusing on the diversity of *bla*_CTX-M_ and *bla*_TEM_ *β*‐lactamase genes. By providing high‐resolution genomic insights into the ESBL determinants circulating within community residents, this study contributes to a more comprehensive understanding of AMR ecology in human reservoirs and informs efforts to mitigate the spread of ESBL‐producing *E. coli* at the community level.

## 2. Materials and Methods

### 2.1. Specimen Collection

Fecal samples provided by community residents were used as specimens in this study. Specimens were collected in Santo Domingo province, located in the subtropical lowlands of the Pacific Coast of Ecuador in August 2023. A total of 55 residents were enrolled in this study. One fecal sample was collected from each participant. The participants ranged in age from 11 to 84 years, with a median age of 44 years, and 27% were male. Multiple participants from the same household were included. All specimens were collected as described previously [22]. The fecal samples collected in this study have also been used for other research purposes and have been reported elsewhere [23, 24].

### 2.2. Ethics Statement

This study was approved by the Ethics Committee of Gifu University (No. 2019‐164) and the Universidad Central del Ecuador (No. LEC IORG 0001932, FWA 2482, IRB 2483 COBI‐AMPHI‐0064‐11). Written informed consent was obtained from all participants and/or their legal guardians. All methods were performed in accordance with relevant guidelines and regulations.

### 2.3. ESBL–*E. coli* Isolation

The fecal specimens were directly inoculated onto MacConkey agar plates supplemented with 4‐*μ*g/mL cefotaxime (CTX) to isolate CTX‐resistant gram‐negative bacteria. Pink colonies that appeared after 24 h of incubation at 37°C were picked and designated as putative CTX‐resistant *E. coli* isolates. Species identification of the obtained isolates was performed using MALDI–TOF MS (Bruker Japan, Kanagawa, Japan).

Isolates identified as *E. coli* were assessed for ESBL production based on the detection of ESBL genes. Briefly, bacterial DNA was extracted by boiling the bacterial suspension in tris (hydroxymethyl) aminomethane–ethylenediaminetetraacetic acid buffer for 10 min. Screening for *bla*_CTX-M_ and *bla*_TEM_ was performed using probe‐based real‐time PCR following previously described methods [25].

### 2.4. Antibiotic Susceptibility of *E. coli* Isolates

ESBL–*E. coli* isolates were evaluated using Eiken Dry Plate DPE1 (Eiken Chemical, Tokyo, Japan), which employs the standard broth microdilution method in accordance with the Clinical and Laboratory Standards Institute (CLSI) guidelines. The antimicrobial panel included 18 agents tested with or without clavulanic acid, which are commonly used in the clinical treatment of bacterial infections and recommended for routine susceptibility testing.

### 2.5. Genome Analysis

Genomic DNA was extracted from *E. coli* isolates using NucleoBond HMW DNA (Macherey‐Nagel, Düren, Germany) according to the manufacturer’s protocol.

Whole‐genome sequences were obtained using a NovaSeq X Plus (Illumina, CA, USA) and a MinION Mk1C sequencer (Oxford Nanopore Technologies, London, UK). Short‐read sequencing was performed using the NovaSeq X Plus high‐throughput sequencing set by a commercial vendor (Genome‐Lead, Takamatsu, Japan). Briefly, genomic DNA libraries were prepared using the QIAseq FX DNA Library Kit (QIAGEN, Hilden, Germany). Libraries were indexed using the QIAseq UDI Y‐Adapter Kit (QIAGEN). Paired‐end sequencing (2 × 150 bp) was performed using Illumina sequencing‐by‐synthesis chemistry. For long‐read sequencing, performed using MinION with an R10.4.1 flow cell, the high molecular weight DNA of each isolate was barcoded using a Rapid Barcoding kit (Oxford Nanopore Technologies). The MinION library was prepared without fragmentation and cleaned using AMPure beads (Beckman Coulter). Reads were collected and processed according to standard quality filtering procedures for downstream analysis. To obtain the complete genome of all tested isolates, a de novo hybrid assembly of both short and long reads was conducted using Unicycler 0.5.0 and Flye 2.9.5‐b1801 with default settings.

**Figure 1:**
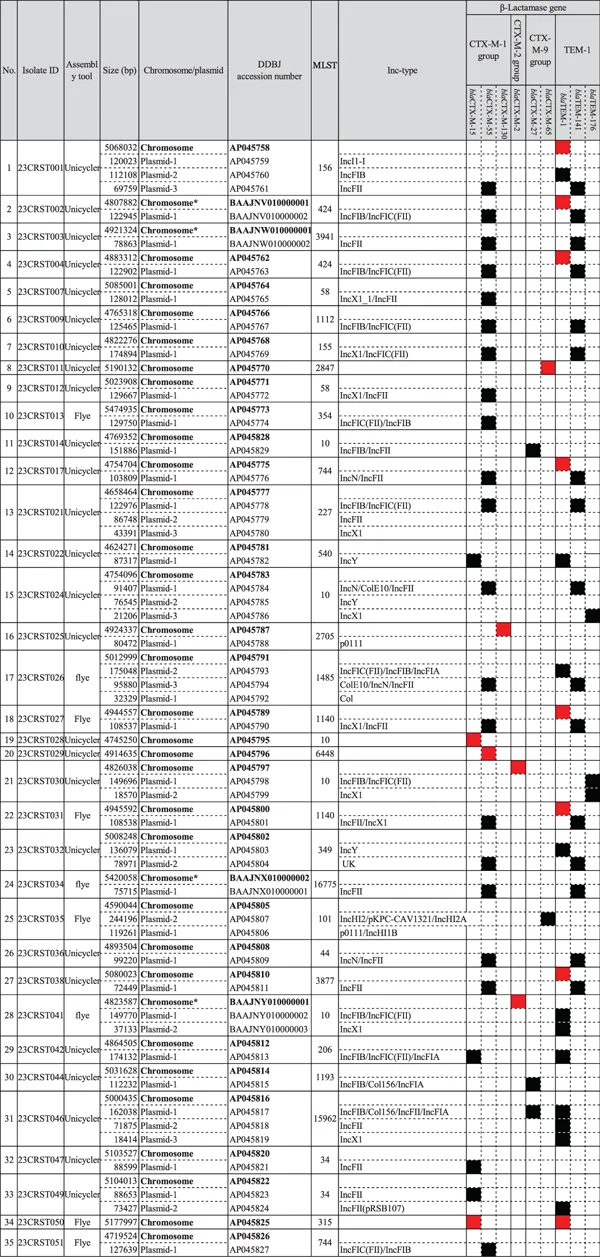
Genome of the ESBL–*E. coli* isolates. Red boxes indicate chromosomal locations. ∗, draft. Bold items relate to chromosomes.

The completely assembled sequences were annotated by uploading FASTA files to the DNA Data Bank of Japan Fast Annotation and Submission Tool v.1.6.0. (https://dfast.ddbj.nig.ac.jp/dfc/) and confirmed using the ResFinder 4.6.0 database (https://cge.food.dtu.dk/services/ResFinder/). Subsequently, the sequences were analyzed using Geneious Prime v.2025.0.3, and *E. coli* plasmid replicons were detected using PlasmidFinder 2.1. (https://cge.food.dtu.dk/services/PlasmidFinder/). The genome sequences generated in this study have been deposited in the DDBJ database under BioProject accession number PRJDB39912. Individual accession numbers for each isolate are listed in Figure 1.

The phylogenetic tree of the isolates was based on core genome single nucleotide polymorphism data generated using CGE CSI Phylogeny 1.4 with default parameters and observed using the Interactive Tree of Life, iTOL v7 (https://itol.embl.de/). *E. coli* strains were assigned to serotype groups using SerotypeFinder 2.0 (https://cge.food.dtu.dk/services/SerotypeFinder/). Phylogenetic grouping of strains was determined using the Clermont phylotyper tool based on *in silico* analysis of whole‐genome sequence data (https://ezclermont.hutton.ac.uk/). Comparative genomic diagrams were generated using Easyfig v2.2.5 and BRIG v0.95 set to default. Genomic annotations were aligned using BLASTn for visualization of synteny and sequence similarity.

## 3. Results

### 3.1. Fecal Carriage of ESBL‐Producing *E. coli* Among Residents

A total of 55 fecal samples were collected from the study participants and cultured on CTX‐MacConkey agar. As summarized in Table 1, *E. coli*–like colonies were detected in 47 samples. Following bacterial identification and PCR screening for ESBL genes, 35 isolates (one per individual) were confirmed to be ESBL‐producing *E. coli*, accounting for 63.6% of the participants.

**Table 1:** Characteristics of ESBL‐producing *E. coli* isolates from resident fecal samples.

Participants	No. of participants with *E. coli*–like colonies on CTX‐McConkey	No. of ESBL‐producing *E. coli* isolates	No. of *E. coli* isolates carrying ESBL gene
55	47	37	35

Genome sequencing and de novo assembly of the 35 ESBL‐producing *E. coli* isolates yielded complete circular genomes for 31 isolates, whereas the remaining four isolates resulted in draft genomes (Figure 1). Although circular chromosomes could not be resolved in these four isolates, circular plasmids harboring ESBL genes were successfully recovered. As shown in Figure 1, genomic analysis revealed that all isolates (100%) harbored *bla*_CTX-M_, whereas 23 isolates (65.7%) carried *bla*_TEM_. Among the *bla*_CTX-M_‐positive strains, 27 (77.1%) belonged to the *bla*_CTX-M-1_ group. All *bla*_TEM_ genes were identified as *bla*_TEM-1_ or its variants. Of the 23 isolates carrying *bla*_TEM-1_, 16 (69.5%) harbored the *bla*_TEM-141_ subtype.

Genomic localization analysis showed that *β*‐lactamase genes were integrated into the chromosome in 14 isolates (40%). Among these, the majority carried *bla*_TEM-1_, with only five strains harboring *bla*_CTX-M_. A total of 30 isolates (85.7%) possessed *β*‐lactamase genes (*bla*_CTX-M_ and/or *bla*_TEM_) on plasmids, and dual localization (both chromosomal and plasmid) was observed in nine isolates (25.7%).

Plasmid incompatibility typing revealed that most *β*‐lactamase‐positive plasmids belonged to the IncF group, with only 7 strains (23.3%) carrying plasmids of non‐IncF types. Remarkably, 22 of 23 IncF plasmids (95.6%) contained multiple IncF replicons.

### 3.2. Genomic Loci of *β*‐Lactamase Genes on Plasmids

**Figure 2:**
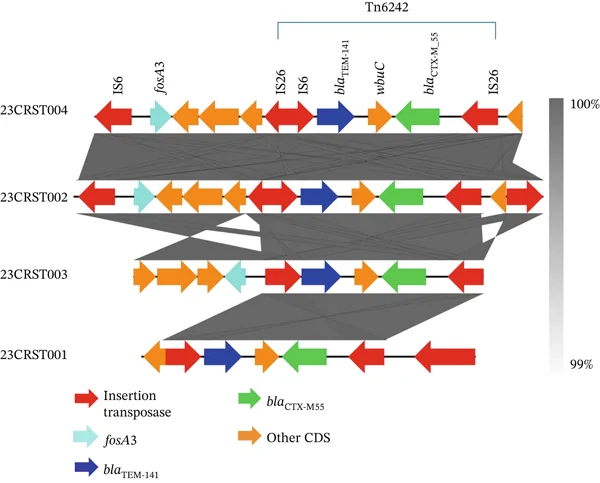
Comparison of the genomic context of blaTEM‐141 and blaCTX‐M‐55 in the IncFII plasmids of ESBL–*E. coli* isolates. The Tn6242‐associated transposase region is indicated as a potential mobile element involved in resistance gene mobilization. Genes are color‐coded by predicted function. Shaded regions between plasmids indicate BLASTn nucleotide sequence similarity. Visualization was generated using Easyfig v2.2.5 based on BLASTn analysis.

Figure 2 illustrates a representative comparison of transposon structures harboring *β*‐lactamase genes on IncF plasmids across multiple isolates. Both *bla*_CTX-M_ and *bla*_TEM_ are embedded within the same transposon, Tn*6242*, flanked by IS*26* elements at both ends. This transposon structure was conserved in 14 of the 16 isolates (87.5%). Furthermore, as shown in Figure S1, the genomic position of this transposon on the plasmid was highly conserved among the isolates.

### 3.3. Genomic Loci of *β*‐Lactamase Genes on Chromosomes

**Figure 3:**
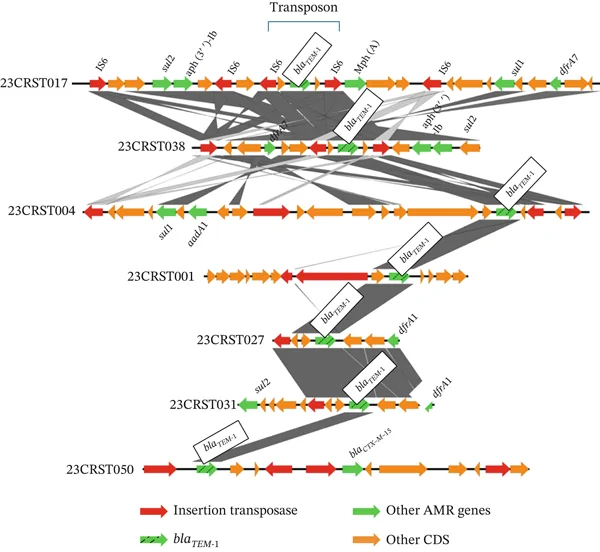
Comparison of the genomic context of blaTEM‐1 in the chromosome of isolates. Genetic chromosome structures containing blaTEM‐1 of 7 isolates are shown. Adjacent genes and mobile genetic elements, including insertion sequences and transposase‐associated regions, are indicated. Genes are color‐coded by predicted function. Shaded blocks between sequences represent BLASTn nucleotide similarity. Visualization was generated using Easyfig v2.2.5 based on BLASTn analysis.

Among the 14 isolates carrying *β*‐lactamase genes on their chromosomes, seven harbored *bla*_TEM_, one of which also carried *bla*_CTX-M_. However, the two genes were located within distinct transposon structures. Figure 3 shows representative chromosomal transposon structures containing *bla*_TEM_. Unlike Tn*6242* on plasmids, these chromosomal transposons lacked *bla*_CTX-M_ and displayed structural variations, such as being flanked by IS*6* elements (members of the IS*26* family) at both ends or missing one IS*6* element. Despite these structural differences, the chromosomal insertion site of *bla*_TEM_ was largely conserved among all *bla*_TEM_‐positive isolates, as depicted in Figure S2.

### 3.4. Antibiotic Resistance Phenotype

**Figure 4:**
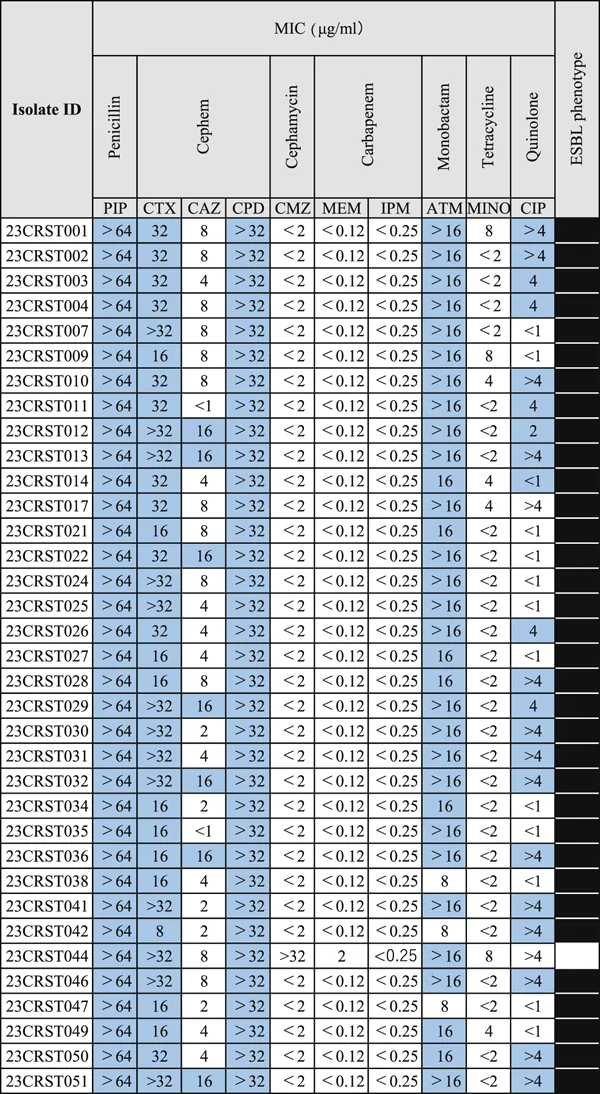
Susceptibility of isolates to antibiotics. The blue square indicates resistance according to CLSI M100 ED35, 2025. ESBL phenotype was determined by a ≥3‐tube decrease in CTX MIC with the addition of clavulanic acid. Black square indicates ESBL‐positive. Abbreviations: ATM, aztreonam; CAZ, ceftazidime; CIP, ciprofloxacin; CMZ, cefmetazole; CPD, cefpodoxime; CTX, cefotaxime; IPM, imipenem; MEM, meropenem; MINO, minocycline; PIP, piperacillin.

Susceptibility profiles of isolated ESBL–*E. coli* isolates in response to various antibiotics are presented in Figure 4. All isolates were phenotypically classified as ESBL‐producing; however, 28 of the 35 isolates (80%) remained susceptible to ceftazidime. No resistance to carbapenems or tetracyclines was observed, and 60% of the isolates were resistant to quinolones.

### 3.5. Phylogenetic Relatedness Among Isolates

**Figure 5:**
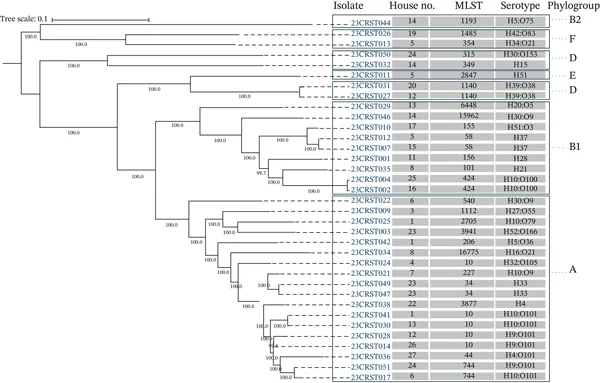
MLST phylogenetic tree of ESBL‐producing *E. coli* isolates obtained from residents in Ecuador. A maximum‐likelihood phylogenetic tree inferred from whole‐genome single‐nucleotide polymorphisms (SNPs) of *E. coli* isolates using CSI Phylogeny is shown. Visualization was performed using the Interactive Tree of Life (iTOL v7). The scale bar represents nucleotide substitutions per site. Bootstrap values indicating node support are shown at the corresponding nodes. House No. denotes the household number of the resident from whom each isolate was obtained.

Figure 5 presents a phylogenetic tree constructed from the whole‐genome sequences of the 35 ESBL‐producing *E. coli* isolates. These isolates were assigned to seven distinct phylogroups, with Phylogroup A being the most prevalent (48.5%; 17 isolates). Notably, this group included five isolates (29.4%) belonging to the globally widespread MLST‐type ST10. However, as detailed in Figure 1, the ESBL gene content of ST10 isolates varied considerably.

Several isolates, including 23CRST004 and 23CRST002, were isolated from different household members but shared the same MLST type (ST424), indicating that they were phylogenetically identical. Similarly, strains 23CRST031 and 23CRST027 were isolated from members of different households, but belonged to the same MLST type (ST1140) and were phylogenetically indistinguishable.

## 4. Discussion

### 4.1. Community Carriage of ESBL‐Producing *E. coli*

ESBL‐producing *E. coli* were highly prevalent in this cohort (63.6%), which is consistent with reports from comparable community‐based studies conducted in diverse geographic settings [8, 23, 26]. This high prevalence may reflect the broader epidemiological landscape in Latin America, where ESBL‐producing organisms are the major components of clinical infections [27]. Such a high prevalence of ESBL‐producing *E. coli* in the intestinal microbiome of residents is likely driven by sustained regional selective pressures that favor the persistence and dissemination of ESBL‐producing *E. coli* within community settings [3]. Among the factors that influence community carriage, the widespread use of antimicrobial agents in agriculture may play a substantial role in promoting the emergence of ESBL‐producing strains and facilitating their transmission to humans through contaminated livestock food products and environmental reservoirs.

The predominant resistance gene background of the *bla*_CTX-M-1_ group revealed by genomic analysis of the isolates appeared to characterize the study area, with the *bla*_CTX-M-1_ group being the major group worldwide and *bla*_CTX-M-15_ thought to have spread in association with the pandemic clone ST131 [28]. *bla*_CTX-M-55_, which was found in the majority of ESBL‐producing *E. coli* strains found in residents of the study area, is closely related to *bla*_CTX-M-15_. However, it is an ESBL that has shown a unique distribution and has been attracting attention for its spread in Asia in recent years [29]. Phylogenetic analysis clearly showed that *bla*_CTX-M-55_ did not spread through specific clones in this region, and horizontal transmission via plasmids and transposons may account for this observation.

TEM enzymes also remain relevant contributors to *β*‐lactam resistance, even in settings where CTX‐M determinants dominate [30, 31]. The finding that most of the isolates identified in this study concurrently harbored *bla*_TEM_ and *bla*_CTX-M_ genes suggests a resistance state in which ESBL‐mediated resistance is superimposed on a pre‐existing foundation of classical *β*‐lactam resistance, thereby conferring broad‐spectrum and potentially high‐level *β*‐lactam resistance with an enhanced capacity for dissemination [32].

### 4.2. Plasmids Carrying ESBL Genes

The finding that IncF group plasmids were the predominant vehicles carrying ESBL genes is consistent with previous reports of ESBL‐producing *E. coli* isolated, albeit in limited numbers, from children and animals in Ecuador [33]. In addition, multireplicon IncF plasmids, particularly those of the FII:FIA:FIBI type, harbor *bla*_CTX-M_ [16, 17, 34, 35]. Our results not only confirm these earlier observations in a community‐resident population but also indicate that IncF group plasmids play a major role in the dissemination of ESBL genes and the spread of ESBL‐producing bacteria in this region of Ecuador.

### 4.3. Conserved IS26‐Flanked ESBL Modules on IncF Plasmids

In this study, *bla*_CTX-M_ and *bla*_TEM_ consistently colocalized within the same IS*26*‐flanked transposon, Tn*6242*, on IncF plasmids, with both the transposon structure and its insertion site on the plasmid backbone conserved in 87.5% of isolates. This high degree of conservation suggests the dissemination of closely related IncF plasmids carrying Tn*6242* within the bacterial population. However, alternative explanations, including preferential insertion into conserved plasmid regions, repeated acquisition of a successful mobile resistance module, or convergent evolution of plasmid architectures, cannot be excluded. Comparative phylogenetic analyses of plasmid backbones would be required to distinguish among these possibilities. Regardless of the underlying mechanism, the observed dissemination pattern is consistent with the established role of IncF plasmids as major vectors for the spread of ESBL genes in clinical settings [32, 36].

The physical linkage of *bla*_CTX-M_ and *bla*_TEM_ within a single mobile genetic element is likely to facilitate coselection under *β*‐lactam antibiotic pressure, thereby enhancing plasmid stability and long‐term persistence [32]. In addition, IS*26* elements flanking Tn*6242* provide a dynamic genetic framework that enables the mobilization and structural rearrangement of resistance gene modules, thereby promoting efficient dissemination while preserving module integrity.

The small number of isolates exhibiting structural divergence, such as the lack of IS*26*, may reflect secondary IS*26*‐mediated rearrangements occurring after plasmid dissemination rather than multiple independent acquisition events.

### 4.4. Chromosomal ESBL Genes

In this study, 40% of the ESBL‐producing *E. coli* isolates harbored *bla*_TEM_ and/or *bla*_CTX-M_ genes on the chromosome. As these genes are typically located on plasmids [37], their chromosomal integration may represent a characteristic feature of ESBL gene distribution among *E. coli* carried by residents in the study area. Considering the role of chromosomally encoded resistance genes in conferring stable resistance [38], the presence of chromosomally integrated *bla*_TEM_ and *bla*_CTX-M_ genes observed here may have implications for the persistence of AMR within community‐associated *E. coli* populations. However, persistence was not directly evaluated in the present study.

In contrast to the conserved Tn*6242* [39], chromosomal *bla*_TEM_ genes were embedded in transposons with structural variability, including IS*6* (IS*26* family)–flanked and truncated configurations. Despite this heterogeneity, the chromosomal insertion site of *bla*_TEM_ was largely conserved among all *bla*_TEM_‐positive isolates. This pattern suggests preferential integration into a limited chromosomal locus followed by secondary IS*26*/IS*6*‐mediated rearrangements that generate derivative structures while maintaining positional stability [40].

The involvement of IS*26* family elements is consistent with their ability to mediate homologous recombination and postintegration remodeling of resistance regions [41]. In contrast, *bla*_CTX-M_ is rarely chromosomal and, when present, is located in a distinct transposon, supporting different mobilization and maintenance pathways for *bla*_TEM_ and *bla*_CTX-M_ [42]. Although limited by sample size and the absence of functional assays, these findings highlight chromosomal resistance loci as potentially important reservoirs of *β*‐lactam resistance. Further studies are needed to determine their stability, persistence, and contribution to resistance dissemination.

### 4.5. Phylogenetic Relatedness Among Isolates

Phylogenetic analysis revealed substantial genetic diversity among the ESBL‐producing *E. coli* isolates, which were distributed across seven phylogroups, with Phylogroup A being predominant, including isolates belonging to the globally disseminated lineage ST10. The marked heterogeneity of ESBL gene content within ST10 indicates that closely related lineages can acquire resistance genes independently, likely via horizontal gene transfer rather than through clonal expansion alone. In contrast, the identification of phylogenetically indistinguishable strains among different household members and across households indicates close genetic relatedness and is consistent with either recent transmission or exposure to common sources. However, because epidemiological linkage data and longitudinal sampling were not available, direct transmission cannot be inferred from the genomic data alone. Taken together, these findings highlight the coexistence of clonal spread and horizontal dissemination of ESBL determinants, a pattern commonly observed in community‐associated ESBL‐producing *E. coli* populations [43–45].

Some limitations should be considered when interpreting our findings. Although whole‐genome sequencing provided high‐resolution characterization of ESBL‐producing *E. coli* and their associated resistance elements, the study was limited to genomic analyses. Consequently, we were unable to assess the expression levels of *β*‐lactamase genes, the stability of plasmid‐borne or chromosomally integrated resistance determinants, or the fitness and transmission consequences associated with different genetic contexts. In addition, the cross‐sectional design and relatively small sample size precluded direct evaluation of temporal persistence and transmission dynamics. Future studies integrating functional assays, longitudinal sampling, and larger cohorts will be valuable for clarifying the biological and epidemiological significance of the genomic features identified here.

## 5. Conclusions

This study demonstrated a high prevalence of community‐associated ESBL‐producing *E. coli* in Ecuador, with all isolates harboring *bla*_CTX-M_ genes, predominantly *bla*_CTX-M-15_ and *bla*_CTX-M-55_. These resistance genes were primarily associated with IncF plasmids carrying conserved IS*26*‐flanked resistance structures, highlighting the major role of mobile genetic elements in the dissemination of *β*‐lactam resistance. Frequent chromosomal integration of *bla*_TEM_ and/or *bla*_CTX-M_ genes further demonstrated the diversity of genomic contexts supporting AMR. Phylogenetic analysis revealed both extensive genetic diversity and evidence of transmission of closely related strains among individuals and households. Together, these findings provide important insights into the molecular epidemiology of community‐associated ESBL‐producing *E. coli* and support the need for continued genomic surveillance to monitor and limit the spread of AMR.

## Funding

This study was supported by Japan Society for the Promotion of Science, 10.13039/501100001691, 23H00446 120229916.

## Conflicts of Interest

The authors declare no conflicts of interest.

## Supporting Information

Additional supporting information can be found online in the Supporting Information section.

## Supporting information


**Supporting Information 1** Figure S1: Comparative genomics of the IncFII plasmids with *bla*_TEM-141_ and *bla*_CTX-M-55_.

**Supporting Information 2** Figure S2: Comparison of the chromosomal locations of *bla*_TEM-1_ among seven bacterial isolates.

## Data Availability

Data derived from this study are available from the corresponding author upon request.

## References

[1] The Lancet , Antimicrobial Resistance: An Agenda for All, Lancet. (2024) 403, no. 10442, 10.1016/S0140-6736(24)01076-6. 38797177

[2] Naghavi M. , Vollset S. E. , Ikuta K. S. , Swetschinski L. R. , Gray A. P. , Wool E. E. , Aguilar G. R. , Mestrovic T. , Smith G. , Han C. , and Hsu R. L. , Global Burden of Bacterial Antimicrobial Resistance 1990–2021: A Systematic Analysis With Forecasts to 2050, Lancet. (2024) 404, no. 10459, 1199–1226, 10.1016/S0140-6736(24)01867-1, 39299261. 39299261 PMC11718157

[3] Ramatla T. , Mafokwane T. , Lekota K. , Monyama M. , Khasapane G. , Serage N. , Nkhebenyane J. , Bezuidenhout C. , and Thekisoe O. , “One Health” Perspective on Prevalence of Co-Existing Extended-Spectrum *β*-Lactamase (ESBL)-Producing Escherichia coli and Klebsiella pneumoniae: A Comprehensive Systematic Review and Meta-Analysis, Annals of Clinical Microbiology and Antimicrobials. (2023) 22, no. 1, 10.1186/s12941-023-00638-3, 37740207. PMC10517531 37740207

[4] Anjum M. F. , Schmitt H. , Börjesson S. , Berendonk T. U. , Donner E. , Stehling E. G. , Boerlin P. , Topp E. , Jardine C. , Li X. , Li B. , Dolejska M. , Madec J. Y. , Dagot C. , Guenther S. , Walsh F. , Villa L. , Veldman K. , Sunde M. , Krzeminski P. , Wasyl D. , Popowska M. , Järhult J. , Örn S. , Mahjoub O. , Mansour W. , Thái Đ. N. , Elving J. , and Pedersen K. , The Potential of Using E. coli as an Indicator for the Surveillance of Antimicrobial Resistance (AMR) in the Environment, Current Opinion in Microbiology. (2021) 64, 152–158, 10.1016/j.mib.2021.09.011, 34739920. 34739920

[5] Raffelsberger N. , Buczek D. J. , Svendsen K. , Småbrekke L. , Pöntinen A. K. , Löhr I. H. , Andreassen L. L. E. , Simonsen G. S. , Norwegian E coli ESBL Study Group , Sundsfjord A. , Gravningen K. , and Samuelsen Ø. , Community Carriage of ESBL-Producing Escherichia coli and Klebsiella pneumoniae: A Cross-Sectional Study of Risk Factors and Comparative Genomics of Carriage and Clinical Isolates, MSphere. (2023) 8, no. 4, e00025-23, 10.1128/msphere.00025-23, 37306968. 37306968 PMC10470604

[6] Olaitan M. O. , Orababa O. Q. , Shittu R. B. , Obunukwu G. M. , Kade A. E. , Arowolo M. T. , Oyediran A. A. , and Yusuff R. A. , Prevalence of ESBL-Producing Escherichia coli in Sub-Saharan Africa: A Meta-Analysis Using a One Health Approach, One Health. (2025) 20, 101090, 10.1016/j.onehlt.2025.101090, 40529906. 40529906 PMC12172985

[7] Kantele A. , Kuenzli E. , Dunn S. J. , Dance D. A. B. , Newton P. N. , Davong V. , Mero S. , Pakkanen S. H. , Neumayr A. , Hatz C. , Snaith A. , Kallonen T. , Corander J. , and McNally A. , Dynamics of Intestinal Multidrug-Resistant Bacteria Colonisation Contracted by Visitors to a High-Endemic Setting: A Prospective, Daily, Real-Time Sampling Study, Lancet Microbe. (2021) 2, no. 4, e151–e158, 10.1016/S2666-5247(20)30224-X, 33821248. 33821248 PMC8009952

[8] Amato H. K. , Loayza F. , Salinas L. , Paredes D. , Garcia D. , Sarzosa S. , Saraiva-Garcia C. , Johnson T. J. , Pickering A. J. , Riley L. W. , Trueba G. , and Graham J. P. , Risk Factors for Extended-Spectrum Beta-Lactamase (ESBL)-Producing E. coli Carriage Among Children in a Food Animal-Producing Region of Ecuador: A Repeated Measures Observational Study, PLoS Medicine. (2023) 20, no. 10, e1004299, 10.1371/journal.pmed.1004299, 37831716. 37831716 PMC10621961

[9] Araque M. and Labrador I. , Prevalence of Fecal Carriage of CTX-M-15 Beta-Lactamase-Producing Escherichia coli in Healthy Children From a Rural Andean Village in Venezuela, Osong Public Health and Research Perspectives. (2018) 9, no. 1, 9–15, 10.24171/J.PHRP.2018.9.1.03, 29503800. 29503800 PMC5831685

[10] Hasan B. , Laurell K. , Rakib M. M. , Ahlstedt E. , Hernandez J. , Caceres M. , and Järhult J. D. , Fecal Carriage of Extended-Spectrum *β*-Lactamases in Healthy Humans, Poultry, and Wild Birds in León, Nicaragua—A Shared Pool of bla_CTX-M_ Genes and Possible Interspecies Clonal Spread of Extended-Spectrum *β*-Lactamases-Producing Escherichia coli, Microbial Drug Resistance. (2016) 22, 682–687, 10.1089/mdr.2015.0323. 27007258

[11] Yu K. , Huang Z. , Xiao Y. , Gao H. , Bai X. , and Wang D. , Global Spread Characteristics of CTX-M-Type Extended-Spectrum *β*-Lactamases: A Genomic Epidemiology Analysis, Drug Resistance Updates. (2024) 73, 101036, 10.1016/j.drup.2023.101036, 38183874. 38183874

[12] Zhang Y. , Lin Y. , Ruan Y. , Yang J. , Holden E. , Felgate H. , Solsona M. , Liu H. , Liang G. , Jiang H. , Webber M. A. , and Zhuo C. , Pivotal Plasmids Drive the Global Spread of CTX-M-27 in *Escherichia coli* , Infection. (2026) 54, no. 1, 315–329, 10.1007/s15010-025-02664-z, 41065996. 41065996 PMC12864245

[13] Zeng S. , Luo J. , Li X. , Zhuo C. , Wu A. , Chen X. , and Huang L. , Molecular Epidemiology and Characteristics of CTX-M-55 Extended-Spectrum *β*-Lactamase-Producing Escherichia coli From Guangzhou, China, Frontiers in Microbiology. (2021) 12, 730012, 10.3389/fmicb.2021.730012, 34707587. 34707587 PMC8542904

[14] Zaatout N. , Bouras S. , and Slimani N. , Prevalence of Extended-Spectrum *β*-Lactamase (ESBL)-Producing Enterobacteriaceae in Wastewater: A Systematic Review and Meta-Analysis, Journal of Water and Health. (2021) 19, no. 5, 705–723, 10.2166/wh.2021.112, 34665765. 34665765

[15] Castanheira M. , Simner P. J. , and Bradford P. A. , Extended-Spectrum *β*-Lactamases: An Update on Their Characteristics, Epidemiology and Detection, JAC-Antimicrobial Resistance. (2021) 3, no. 3, dlab092, 10.1093/jacamr/dlab092, 34286272. 34286272 PMC8284625

[16] Rozwandowicz M. , Brouwer M. S. M. , Fischer J. , Wagenaar J. A. , Gonzalez-Zorn B. , Guerra B. , Mevius D. J. , and Hordijk J. , Plasmids Carrying Antimicrobial Resistance Genes in Enterobacteriaceae, Journal of Antimicrobial Chemotherapy. (2018) 73, no. 5, 1121–1137, 10.1093/jac/dkx488. 29370371

[17] Mathers A. J. , Peirano G. , and Pitout J. D. D. , The Role of Epidemic Resistance Plasmids and International High-Risk Clones in the Spread of Multidrug-Resistant Enterobacteriaceae, Clinical Microbiology Reviews. (2015) 28, no. 3, 565–591, 10.1128/CMR.00116-14, 25926236. 25926236 PMC4405625

[18] Zhang M. , Qin X. , Ding B. , Shen Z. , Sheng Z. , Wu S. , Yang Y. , Xu X. , Hu F. , Wang X. , and Zhang Y. , Antibiotic Exposure During the Preceding Six Months Is Related to Intestinal ESBL-Producing Enterobacteriaceae Carriage in the Elderly, Antibiotics. (2022) 11, 10.3390/antibiotics11070953, 35884207. PMC9312271 35884207

[19] Che Y. , Yang Y. , Xu X. , Brinda K. , Polz M. F. , Hanage W. P. , and Zhang T. , Conjugative Plasmids Interact With Insertion Sequences to Shape the Horizontal Transfer of Antimicrobial Resistance Genes, Proceedings of the National Academy of Sciences. (2021) 118, no. 6, e2008731118, 10.1073/pnas.2008731118, 33526659. PMC8017928 33526659

[20] Salinas L. , Loayza F. , Cárdenas P. , Saraiva C. , Johnson T. J. , Amato H. , Graham J. P. , and Trueba G. , Environmental Spread of Extended Spectrum Beta-Lactamase (ESBL) Producing Escherichia coli and ESBL Genes Among Children and Domestic Animals in Ecuador, Environmental Health Perspectives. (2021) 129, no. 2, 27007, 10.1289/EHP7729, 33617318. 33617318 PMC7899495

[21] Montero L. , Irazabal J. , Cardenas P. , Graham J. P. , and Trueba G. , Extended-Spectrum Beta-Lactamase Producing-Escherichia coli Isolated From Irrigation Waters and Produce in Ecuador, Frontiers in Microbiology. (2021) 12, 709418, 10.3389/fmicb.2021.709418, 34671324. 34671324 PMC8521160

[22] Yamamoto Y. , Calvopina M. , Izurieta R. , Villacres I. , Kawahara R. , Sasaki M. , and Yamamoto M. , Colistin-Resistant Escherichia coli With mcr Genes in the Livestock of Rural Small-Scale Farms in Ecuador, BMC Research Notes. (2019) 12, no. 1, 10.1186/s13104-019-4144-0, 30832731. PMC6399824 30832731

[23] Yamamoto Y. , Hoang H. T. T. , Le Y. H. , Appiah-Kwarteng C. , Khong D. T. , and Nguyen T. N. , Multinational Comparison of the Detection of Extended-Spectrum Beta-Lactamase Genes in Healthy Resident Feces, Microbiology Spectrum. (2025) 13, no. 6, e0292024, 10.1128/spectrum.02920-24, 40304472. 40304472 PMC12131811

[24] Thanh Hoang H. T. , Yamamoto M. , Calvopina M. , Bastidas-Caldes C. , Khong D. T. , Nguyen T. N. , and Yamamoto Y. , Ban of Colistin Livestock Feed Supplementation Decreased the Prevalence of Colistin-Resistant Escherichia coli in Ecuador, Journal of Infection and Chemotherapy. (2025) 31, no. 4, 102682, 10.1016/j.jiac.2025.102682, 40113139. 40113139

[25] Le Y. H. , Hoang H. T. T. , Khong D. T. , Nguyen T. N. , Que T. A. , Pham D. T. , Tanaka K. , and Yamamoto Y. , Contamination of Retail Market Meat With Extended-Spectrum Beta-Lactamase Genes in Vietnam, International Journal of Food Microbiology. (2025) 430, 111061, 10.1016/j.ijfoodmicro.2025.111061, 39827751. 39827751

[26] Bastidas-Caldes C. , Cisneros-Vásquez E. , Zambrano A. , Mosquera-Maza A. , Calero-Cáceres W. , Rey J. , Yamamoto Y. , Yamamoto M. , Calvopiña M. , and de Waard J. H. , Co-Harboring of Beta-Lactamases and mcr-1 Genes in Escherichia coli and Klebsiella pneumoniae From Healthy Carriers and Backyard Animals in Rural Communities in Ecuador, Antibiotics. (2023) 12, no. 5, 10.3390/antibiotics12050856, 37237759. PMC10215259 37237759

[27] Gozzer E. , Becerra-Chauca N. , Abba-Aji M. , Wirtz V. J. , Cordoba G. , Canchihuamán F. et al., Antimicrobial Resistance Interventions in Latin America and the Caribbean: A Scoping Review of Reported Interventions Between 2018–2024, Antimicrobial Resistance and Infection Control. (2025) 14, 10.1186/s13756-025-01629-z, 41220030. PMC12607118 41220030

[28] Bevan E. R. , Jones A. M. , and Hawkey P. M. , Global Epidemiology of CTX-M *β*-Lactamases: Temporal and Geographical Shifts in Genotype, Journal of Antimicrobial Chemotherapy. (2017) 72, no. 8, 2145–2155, 10.1093/jac/dkx146, 28541467. 28541467

[29] Yang J. T. , Zhang L. J. , Lu Y. , Zhang R. M. , and Jiang H. X. , Genomic Insights Into Global bla_CTX-M_-_55_-Positive Escherichia coli Epidemiology and Transmission Characteristics, Microbiology Spectrum. (2023) 11, no. 4, e0108923, 10.1128/spectrum.01089-23, 37358409. 37358409 PMC10434037

[30] Hernández-Alomía F. , Bastidas-Caldes C. , Zambrano A. , Narváez M. , and Castillejo P. , Molecular Characterization of Antimicrobial Resistance Genes and Plasmid Profiles in Enterobacterales Isolated From Urinary Tract Infections in Rural Outpatient Women in Otavalo, Ecuador, BMC Infectious Diseases. (2025) 25, no. 1, 10.1186/s12879-025-11604-z, 41131447. PMC12548206 41131447

[31] Elsayed A. G. A. , Badr D. F. , El Kheir N. Y. A. , Zaki M. E. S. , Mossad A. E. M. , and Mahmoud E. M. F. , Prevalence of Extended-Spectrum Beta-Lactamase and Molecular Detection of blaTEM, blaSHV, and blaCTX-M Genotypes Among Gram-Negative Bacilli Isolates From Hospital Acquired Infections in Pediatrics, One Institutional Study, Italian Journal of Pediatrics. (2024) 50, no. 1, 10.1186/s13052-024-01599-9, 38402215. PMC10893665 38402215

[32] Carattoli A. , Plasmids and the Spread of Resistance, International Journal of Medical Microbiology. (2013) 303, no. 6-7, 298–304, 10.1016/j.ijmm.2013.02.001. 23499304

[33] Salinas L. , Cárdenas P. , Graham J. P. , and Trueba G. , IS 26 Drives the Dissemination of bla_CTX-M_ Genes in an Ecuadorian Community, Microbiology Spectrum. (2024) 12, no. 1, e02504–e02523, 10.1128/spectrum.02504-23, 38088550. 38088550 PMC10783052

[34] Stephens C. , Arismendi T. , Wright M. , Hartman A. , Gonzalez A. , Gill M. , Pandori M. , and Hess D. , F Plasmids Are the Major Carriers of Antibiotic Resistance Genes in Human-Associated Commensal *Escherichia coli* , MSphere. (2020) 5, no. 4, 10–1128, 10.1128/msphere.00709-20, 32759337. PMC7407071 32759337

[35] Villa L. , García-Fernández A. , Fortini D. , and Carattoli A. , Replicon Sequence Typing of IncF Plasmids Carrying Virulence and Resistance Determinants, Journal of Antimicrobial Chemotherapy. (2010) 65, no. 12, 2518–2529, 10.1093/jac/dkq347. 20935300

[36] D’Andrea M. M. , Arena F. , Pallecchi L. , and Rossolini G. M. , CTX-M-Type *β*-Lactamases: A Successful Story of Antibiotic Resistance, International Journal of Medical Microbiology. (2013) 303, no. 6-7, 305–317, 10.1016/j.ijmm.2013.02.008, 23490927. 23490927

[37] Moxon C. A. and Paulus S. , Beta-Lactamases in Enterobacteriaceae Infections in Children, Journal of Infection. (2016) 72, S41–S49, 10.1016/j.jinf.2016.04.021. 27180312

[38] Zongo P. D. , Cabanel N. , Royer G. , Depardieu F. , Hartmann A. , Naas T. , Glaser P. , and Rosinski-Chupin I. , An Antiplasmid System Drives Antibiotic Resistance Gene Integration in Carbapenemase-Producing *Escherichia coli* Lineages, Nature Communications. (2024) 15, no. 1, 10.1038/s41467-024-48219-y, 38750030. PMC11096173 38750030

[39] Chowdhury P. R. , McKinnon J. , Liu M. , and Djordjevic S. P. , Multidrug Resistant Uropathogenic *Escherichia coli* ST405 With a Novel, Composite IS26 Transposon in a Unique Chromosomal Location, Frontiers in Microbiology. (2019) 9, 10.3389/fmicb.2018.03212, 30671039. PMC6331395 30671039

[40] Varani A. , He S. , Siguier P. , Ross K. , and Chandler M. , The IS6 Family, a Clinically Important Group of Insertion Sequences Including IS26, Mobile DNA. (2021) 12, no. 1, 10.1186/s13100-021-00239-x, 33757578. PMC7986276 33757578

[41] Harmer C. J. , Moran R. A. , and Hall R. M. , Movement of IS26-Associated Antibiotic Resistance Genes Occurs via a Translocatable Unit That Includes a Single IS26 and Preferentially Inserts Adjacent to Another IS26, MBio. (2014) 5, no. 5, 10–1128, 10.1128/mBio.01801-14. PMC4196232 25293759

[42] Partridge S. R. , Kwong S. M. , Firth N. , and Jensen S. O. , Mobile Genetic Elements Associated With Antimicrobial Resistance, Clinical Microbiology Reviews. (2018) 31, no. 4, 10–1128, 10.1128/CMR.00088-17, 30068738. PMC6148190 30068738

[43] Yartey S. N. A. , Kungu F. , Asantewaa A. A. , and Donkor E. S. , Extended Spectrum Beta-Lactamase-Producing Bacterial Clones in West Africa: A Systematic Review and Meta-Analysis From a One Health Perspective, Scientific Reports. (2025) 15, no. 1, 29625, 10.1038/s41598-025-10695-7, 40796576. 40796576 PMC12344016

[44] Hayer J. , Salgado-Caxito M. , Opazo-Capurro A. , Muñoz P. G. , Millán J. , Piñeiro A. , Munita J. M. , Rivas L. , and Benavides J. A. , Multiple Clonal Transmissions of Clinically Relevant Extended-Spectrum Beta-Lactamase–Producing Escherichia coli Among Livestock, Dogs, and Wildlife in Chile, Journal of Global Antimicrobial Resistance. (2023) 34, 247–252, 10.1016/j.jgar.2023.07.009, 37463613. 37463613

[45] Pietsch M. , Eller C. , Wendt C. , Holfelder M. , Falgenhauer L. , Fruth A. , Grössl T. , Leistner R. , Valenza G. , Werner G. , Pfeifer Y. , and RESET Study Group , Molecular Characterisation of Extended-Spectrum *β*-Lactamase (ESBL)-Producing Escherichia coli Isolates From Hospital and Ambulatory Patients in Germany, Veterinary Microbiology. (2017) 200, 130–137, 10.1016/j.vetmic.2015.11.028, 26654217. 26654217

